# The Impact of Levetiracetam on Various Symptoms of Morphine Dependence in Mice

**DOI:** 10.3390/life16081274

**Published:** 2026-07-31

**Authors:** Piotr Listos, Krzysztof Fronc, Antonina Mazur, Mateusz Bosiacki, Jolanta Kotlińska, Tymoteusz Słowik, Joanna Listos

**Affiliations:** 1 Department of Pathomorphology and Forensic Medicine, Faculty of Veterinary Medicine, University of Life Sciences in Lublin, ul. Głęboka 30, 20-612 Lublin, Poland; 2 Department of Pharmacology and Pharmacodynamics, Medical University of Lublin, Chodźki 4a St., 20-093 Lublin, Poland; krzysztof.michal.fronc@gmail.com (K.F.); tosiag@o2.pl (A.M.); jolanta.kotlinska@umlub.edu.pl (J.K.); joanna.listos@umlub.edu.pl (J.L.); 3 Department of Biochemistry, Pomeranian Medical University, Powstańców Wlkp. 72 Av., 70-111 Szczecin, Poland; mateusz.bosiacki@pum.edu.pl; 4 Experimental Medicine Center, Medical University of Lublin, Jaczewskiego 8d St., 20-090 Lublin, Poland; tymoteusz.slowik@umlub.edu.pl

**Keywords:** morphine dependence, morphine tolerance, levetiracetam, behavioral sensitization, hot plate test, withdrawal signs

## Abstract

Levetiracetam (LEV) is an antiepileptic drug and is used in the management of various types of seizures and other clinical conditions, including anxiety and neuropathic pain. Its unique mechanism primarily involves selective interaction with synaptic vesicle protein 2A (SV2A), which stabilizes synaptic function and the release of neurotransmitters. The objective of the present study was to evaluate LEV activity in morphine (MPH) dependence. In mice, LEV was used at doses ranging from 31.25 to 125 mg/kg, depending on the experimental paradigm. It was studied in three schedules: (1) MPH tolerance to antinociceptive effects (the hot plate test—HPT); (2) MPH withdrawal signs, manifested as jumps, induced by naloxone (NAL)—2 mg/kg, i.p.; (3) MPH sensitization to locomotor activity. In this study, LEV reduced MPH tolerance to analgesic effects. Moreover, LEV diminished the intensity of NAL-precipitated MPH withdrawal signs. This drug was also effective in attenuating MPH-induced sensitization. These findings suggest that LEV may modulate behavioral adaptations associated with repeated MPH exposure. However, further mechanistic studies are required to identify the molecular and neurochemical pathways involved in these effects.

## 1. Introduction

Levetiracetam (LEV) is an antiepileptic drug and was implemented in patient care in 2000. Chemically, it is classified as a pyrrolidone derivative and is the S-enantiomer of α-ethyl-2-oxo-1-pyrrolidineacetamide. LEV is primarily utilized to treat different types of seizures, including focal, myoclonic, and generalized tonic–clonic seizures. Its broad therapeutic spectrum and favorable side effect profile make it a cornerstone in epilepsy management. Nowadays, LEV is considered an effective drug in epilepsy, as both a monotherapy and add-on therapy [1]. Because LEV shows diverse pleiotropic effects, it is also increasingly used to treat other conditions, such as bipolar disorder and other mood disorders. In some studies, its effectiveness has been observed in neuropathic pain and in the attenuation of symptoms related to addictive substances [2,3]. It has also shown activity in experimental models of neuropathic pain [4] and inflammatory pain [5] in mice. There are also data showing that LEV-induced analgesia was effective in reducing cancer pain that persisted despite standard therapy [2]. Moreover, its beneficial activity was confirmed in a model of alcohol dependence [6].

LEV’s mechanism is unusual compared with that of other antiepileptics. It selectively and reversibly binds synaptic vesicle protein 2A (SV2A), which is found in abundance in synaptic terminals and participates in the modulation of neurotransmitter release and synaptic transmission [7,8]. Through SV2A modulation, LEV may stabilize synaptic function and reduce excessive neuronal excitability [9,10,11]. Additional effects on calcium channels and intracellular calcium signaling have also been reported [12,13,14,15,16,17]. In contrast, LEV does not seem to operate through classical mechanisms typical of many antiepileptic drugs, such as the modulation of voltage-dependent sodium channels [14].

Morphine (MPH), an important drug for the treatment of pain, is also one of the drugs with the greatest risks of dependence [18]. MPH activates μ-opioid receptors in the ventral tegmental area (VTA) on GABAergic terminals, which contributes to MPH’s rewarding properties. The activation of μ-opioid receptors inhibits GABA release, which increases dopamine release in the nucleus accumbens. This produces MPH’s euphoric effect and contributes to dependence [19]. Morphine dependence is manifested by characteristic unpleasant symptoms referred to as MPH withdrawal signs. Another important parameter is MPH tolerance, identified as a necessity for dose escalation to achieve the same pharmacological effect [20]. A further parameter associated with dependence is behavioral sensitization, which develops after the repeated, intermittent administration of an addictive substance, including MPH. It is expressed as an enhanced pharmacological response to the drug after a drug-free period and is considered to reflect drug-seeking behavior and relapse vulnerability [21]. The current pharmacotherapy for MPH dependence remains insufficiently effective, and, despite numerous studies, no innovative drugs have been introduced for many years. Interestingly, some gabapentinoids have shown promising effects in the modulation of opioid dependence-related symptoms. For example, Vashchinkina et al. [22] demonstrated that pregabalin could attenuate several MPH-related effects in mice, including NAL-precipitated withdrawal symptoms, hyperlocomotion, and MPH self-administration, while Pysiewicz et al. [23] showed that topiramate reduced MPH tolerance and withdrawal. Therefore, new, innovative ideas are extremely important in the search for pharmacological tools for the management of opioid dependence.

Taking into consideration the mechanism of action of LEV, it may be hypothesized that LEV could be effective in MPH dependence. The analysis of existing data shows that co-administration of LEV with MPH is relatively safe and that clinical interactions between these drugs are not significant. Treatment of epileptic seizures with subcutaneous LEV in palliative patients co-treated with MPH does not evoke significant alterations in the antiseizure effect of LEV, its median dose, the median osmolarity of the infusion solution, or the infusion rate [24]. LEV does not affect cytochrome P450, and its metabolism in the liver is poor. Predominantly, it is excreted by the kidneys in unchanged form. On the other hand, MPH pharmacokinetics is associated with uridine 5′-diphosphoglucuronosyltransferases in the liver. Thus, there is a relatively low risk of pharmacokinetic interaction between these drugs [25]. LEV, instead of phenobarbital, was effective in attenuating neonatal abstinence syndrome, ref. [26] which supports the lack of pharmacokinetic interactions between these compounds. The diverse mechanisms of both drugs support the hypothesis that mainly pharmacodynamic interactions between both drugs may occur. In fact, their combination at higher doses may increase neurological side effects, such as dizziness, drowsiness, or confusion; however, these adverse effects can be attenuated by dose reduction. This suggests that the potential relationship between both drugs is worth studying.

In this paper, we present a series of behavioral experiments performed in mice showing the effectiveness of LEV in selected experimental paradigms of the state of dependence to MPH, including tolerance to the antinociceptive effect of MPH, MPH withdrawal manifested as jumping behavior, and MPH-induced locomotor sensitization. The findings support further preclinical investigation of LEV in behavioral models related to MPH dependence.

## 2. Materials and Methods

### 2.1. General Experimental Conditions

The study was conducted in mice (Swiss, male). The weight of animals was between 20 and 25 g. Mice were kept 4–5/housing cage and exposed to a normal night/day cycle. Environmental temperature was 22 ± 1 °C. A commonly used diet (Murigran, Motycz, Poland) and water were provided without restrictions. Before the study, the animals underwent a 7-day acclimatization period. Next, mice were randomly assigned to groups. Behavioral procedures were performed by investigators blinded to the study. The experiments were performed in a well-lit room with reduced external noise. The total number of all animals taken in all experimental protocols was *N* = 440, with 8–10 animals assigned to each experimental group. This group size was selected based on previous experience with experimental procedures and was chosen to ensure reliable behavioral assessment while minimizing animal numbers, in accordance with the principles of the 3Rs. No formal a priori power calculation was performed before the study. The total number of animals was relatively high because the study included several independent behavioral protocols assessing MPH tolerance, NAL-precipitated withdrawal signs, and MPH-induced behavioral sensitization, including separate groups for the expression and acquisition phases, different doses of LEV, and appropriate control groups.

All procedures were conducted according to the National Institutes of Health Guidelines for the Care and Use of Laboratory Animals and to the European Community Council Directive for the Care and Use of Laboratory Animals, and were approved by the Local Ethics Committee for Animal Experiments in Lublin, operating at the University of Life Sciences in Lublin, Poland (No 20/2019, No 33/2019).

### 2.2. Chemical Compounds

The drugs mentioned below were given in all experiments: LEV (Keppra, UCB Pharma SA, Braine-l’Alleud, Belgium, pharmaceutical grade; 250 mg LEV per film-coated tablet; lot no. 209944), MPH hydrochloride trihydrate (Cosmetic Pharma, Kraków, Poland, lot no. 180578; purity: 98%), NAL hydrochloride (Sigma Aldrich, substance, St. Louis, MO, USA, lot no. 180578; purity: ≥98%). All substances were freshly prepared before use and were intraperitoneally (i.p.) injected (volume 10 mL/kg). LEV was triturated and suspended in 0.5% methylcellulose solution. NAL hydrochloride and MPH hydrochloride were dissolved in saline. The final concentrations were adjusted according to the administered dose and injection volume. LEV’s doses were expressed as LEV, whereas doses of MPH and NAL were expressed as salt forms. There were two control groups in the preliminary study: (1) receiving saline; (2) receiving 0.5% methylcellulose solution. At the appropriate time point before testing, mice received an equal volume of the respective treatment solution. No differences were observed between these groups; therefore, only one control group is shown in the figures, namely the methylcellulose group.

LEV was used at doses of 31.25, 62.5, and 125 mg/kg. Dose selection was guided by the literature data [27,28] and preliminary experiments performed in our laboratory. These results are presented in Supplementary File S1. Each group consisted of 8–10 mice. Furthermore, technical data are provided in a table in Supplementary File S2.

### 2.3. Behavioral Procedures

#### 2.3.1. MPH Tolerance

The MPH tolerance to antinociception was studied in the hot plate test (HPT) [23,29,30]. The HPT was performed from 9.00 a.m. to 2.00 p.m. The experimental chamber was a round metal plate (diameter: 20 cm) heated to 55 ± 0.5 °C with transparent walls (height: 18 cm). Before testing, animals were placed (60 min) in the experimental room for 60 min. The mouse was then placed individually on a round plate. After every measurement, the plate surface was cleaned with 70% ethanol. In this test, the time(s) spent by each mouse on the plate was measured until the first reaction of the mouse to the thermal stimulus, defined as either paw licking, hind paw withdrawal, or jumping. The maximum observation time was set to 60 s; however, no animal reached this cut-off time during the experiment. To induce tolerance, mice received MPH (10 mg/kg) twice daily for 7 days. The HPT was performed on days 1 and 7.

**Scheme 1:**
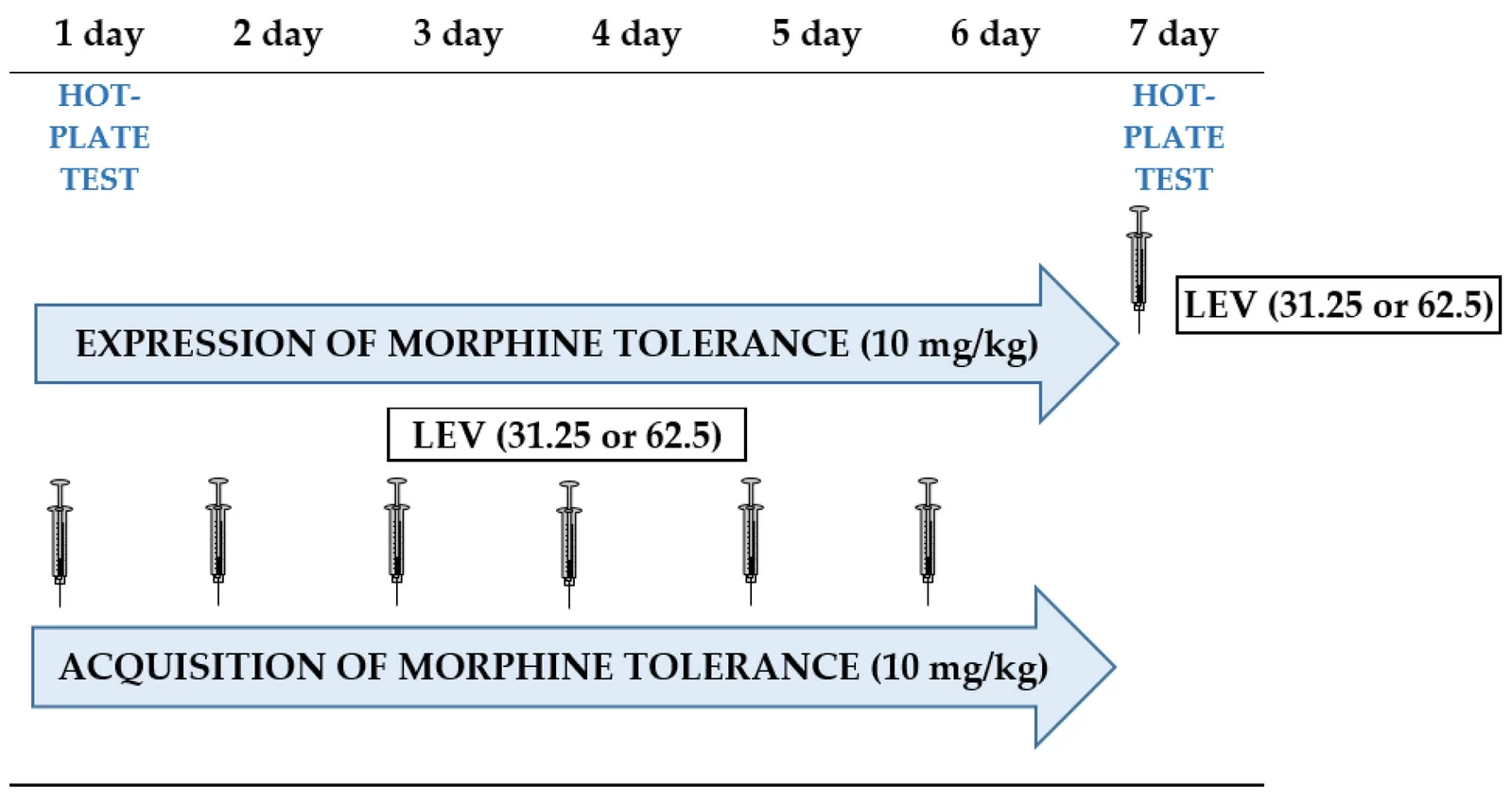
Drug administration in the procedure of MPH tolerance.

The effect of LEV on MPH tolerance was evaluated in two protocols: expression and acquisition. In the expression protocol, a single dose of LEV was administered on day 7, 30 min before MPH injection. The HPT was performed 30 min after MPH administration, and the response latency to the nociceptive stimulus was recorded in seconds. In the acquisition protocol, LEV was administered once daily for 6 days, from day 1 to day 6 of the experiment, 30 min before MPH injection. On day 7, only MPH was administered, and the HPT was performed 30 min later. This procedure is illustrated in Scheme 1. 

#### 2.3.2. NAL-Induced MPH Withdrawal Signs

To induce MPH dependence in mice, an experimental procedure developed in our Department was used [31,32]. According to this protocol, increasing MPH doses, ranging from 10 to 50 mg/kg, were injected for 8 days, twice daily. On the 9th day (morning), the next MPH dose was injected (50 mg/kg). Sixty minutes later, NAL (2.0 mg/kg) was given to evoke MPH withdrawal syndrome. More intensive MPH exposure protocols using increasing doses over shorter periods have also been described by other authors [33]. After NAL injection, the mice were placed individually into glass cylinders (10 L), and jumps were recorded over a 30-min period by an evaluator blinded to the study. A “jump” was considered an episode when the animal simultaneously removed all four paws from the floor. Although other somatic signs are commonly assessed in opioid withdrawal paradigms, jumps were selected in the present study as the principal endpoint because of their high reliability, clear manifestation, and suitability for quantitative pharmacological assessment.

**Scheme 2:**
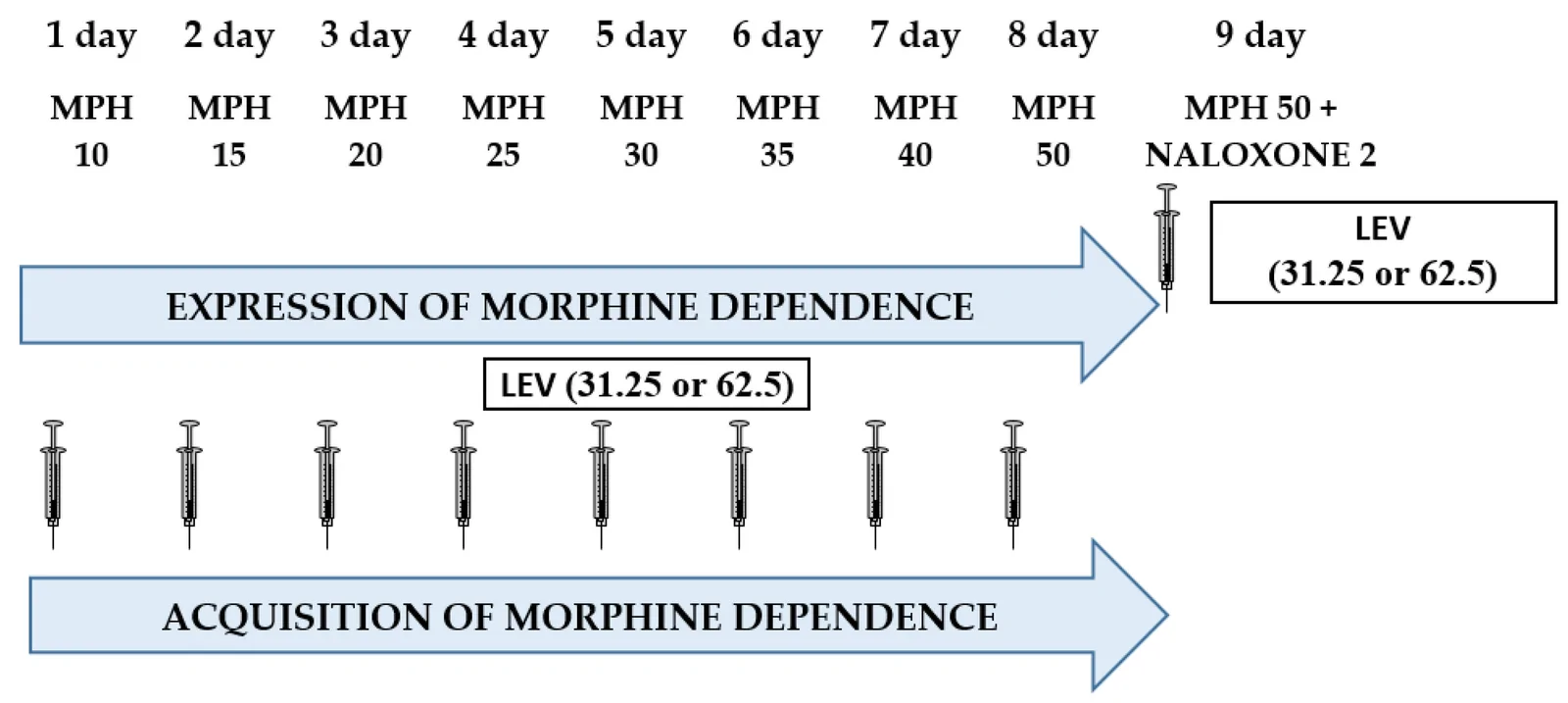
Drug administration in the procedure of MPH dependence.

To evaluate the effect of LEV on MPH withdrawal signs, two experimental protocols were applied. In the first protocol, the influence of a single dose of LEV was examined by administering LEV 20 min before MPH on day 9 of the study. Next, one hour after the MPH injection, NAL was used, and the animal’s behavior was observed. In the second protocol, LEV was given repeatedly for 8 days (once daily), 20 min before the MPH injection, while on day 9 of the study, mice received MPH alone, followed 1 h later by NAL administration and behavioral assessment. This procedure is illustrated in Scheme 2.

#### 2.3.3. MPH Sensitization

MPH-induced sensitization was studied using the method of Kuribara [34], as modified by others [35,36]. For the measurement of mice’s locomotor activity, activity meters (Multiserv, Lublin, Poland) were applied. They consisted of round, metal cages, 32 cm in diameter, with photoresistors installed in the walls. The photoresistors were crossed by two perpendicular light beams positioned 1 cm above the floor. When a moving animal crossed a beam, the change in the photoresistor signal was recorded by the appropriate counter as one movement. The value recorded by the counter indicated spontaneous locomotor activity in mice [37]. Behavioral testing was performed under constant lighting conditions. Before the experiments, animals were habituated to the room for 30 min. After each trial, the actimeter chamber was sanitized with 70% alcohol. Behavioral sensitization was obtained by sporadic MPH administration (10 mg/kg), injected every 72 h on days 1, 4, 7, 10, and 13. After MPH administration, the animals were put into locomotor activity chambers, and cumulative locomotor activity was measured for 1 h. Seven days later, on day 20, the animals received an MPH dose (10 mg/kg)—the challenge dose—and their locomotion was assessed. The same animals assigned to each experimental group were used throughout the entire sensitization protocol, including the assessments performed on days 1 and 20. No additional animals were introduced on day 20.

**Scheme 3:**
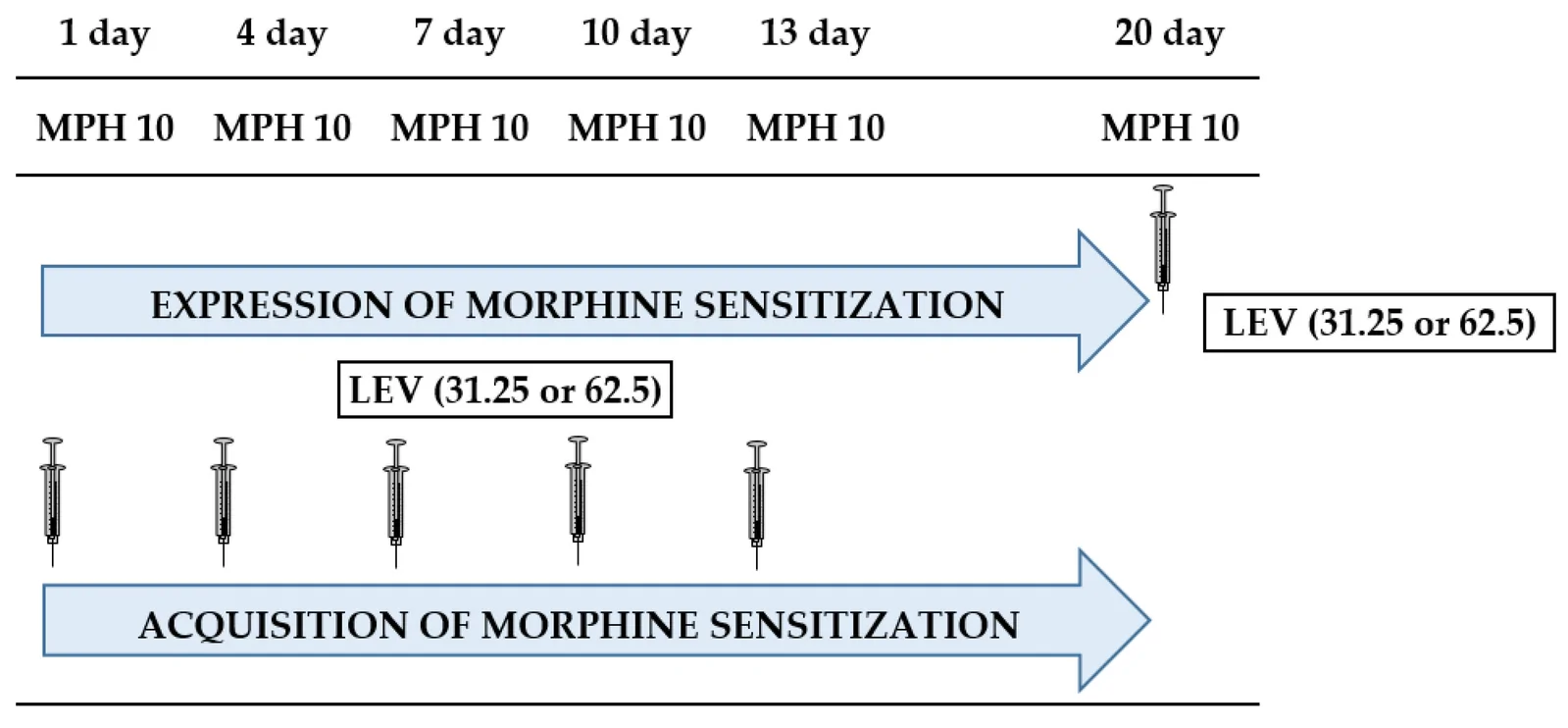
Drug administration in the procedure of MPH sensitization.

Similarly, the effect of LEV on MPH sensitization was studied in two experimental protocols. Firstly, the influence of LEV in the expression model was studied by single-dose administration (on day 20), 30 min before MPH injection. Next, the animals were put into the locomotor activity chambers for 60 min to measure their activity. Secondly, LEV was administered sporadically in the acquisition model, 30 min before MPH injection on days 1, 4, 7, 10, and 13. On day 20, mice received an MPH challenge dose without LEV. Subsequently, the mice were placed in activity meters for 1 h to measure their locomotor activity. This procedure is illustrated in Scheme 3.

### 2.4. The Statistical Calculations

GraphPad Prism 8.3.0 Software (Boston, MA, USA) was used for statistical analysis. When the data followed a normal distribution as assessed by the Shapiro–Wilk test, ANOVA was performed. In the MPH tolerance and MPH-induced sensitization experiments, the data were analyzed as unpaired group-level observations rather than as paired repeated measurements of individual animals. Therefore, comparisons between days 1 and 7 in the MPH tolerance experiment and between days 1 and 20 in the sensitization experiment were not treated as paired individual measurements, and repeated-measures ANOVA was not applied. Instead, ordinary two-way ANOVA followed by Tukey’s multiple comparisons test with adjusted *p*-values was applied. NAL-precipitated MPH withdrawal signs were analyzed by one-way ANOVA according to the Shapiro–Wilk test; the data were approximately normally distributed. Post hoc comparisons were also performed using Tukey’s multiple comparisons test with adjusted *p*-values.

The results of the MPH tolerance experiment are presented as mean time (s) ± SEM (standard error of the mean). In the NAL-precipitated withdrawal test, jumps were counted over a 30-min period and are presented as the mean number of jumps ± SEM. MPH-induced sensitization was assessed based on locomotor activity and is shown as the mean number of movements ± SEM. In all procedures, individual data points were excluded from the analysis only in cases of identifiable technical or procedural errors. In total, 44 individual data points were excluded. All remaining data were included in the final statistical analysis. Differences were considered significant when *p* < 0.05. The number of animals (*N*) in each group was 8–10 mice. Additional data are presented in Supplementary File S2.

## 3. Results

### 3.1. The Impact of LEV (31.25; 62.5 mg/kg, i.p.) on MPH (10 mg/kg, i.p.) Tolerance—Expression Model

Using a two-way ANOVA, statistical changes were demonstrated in mice treated with both single and chronic doses of MPH, assessed on the 1st and 7th days, compared with control animals. The following were observed: a day effect (F(1,36) = 6.297; *p* = 0.0167), a drug effect (F(1,36) = 25.06; *p* < 0.0001), and an interaction (F(1,36) = 5.791; *p* = 0.0214). Moreover, one-way ANOVA analysis showed statistical differences in the reaction time of mice on the 7th day (F(5,52) = 22.08; *p* < 0.0001).

**Figure 1:**
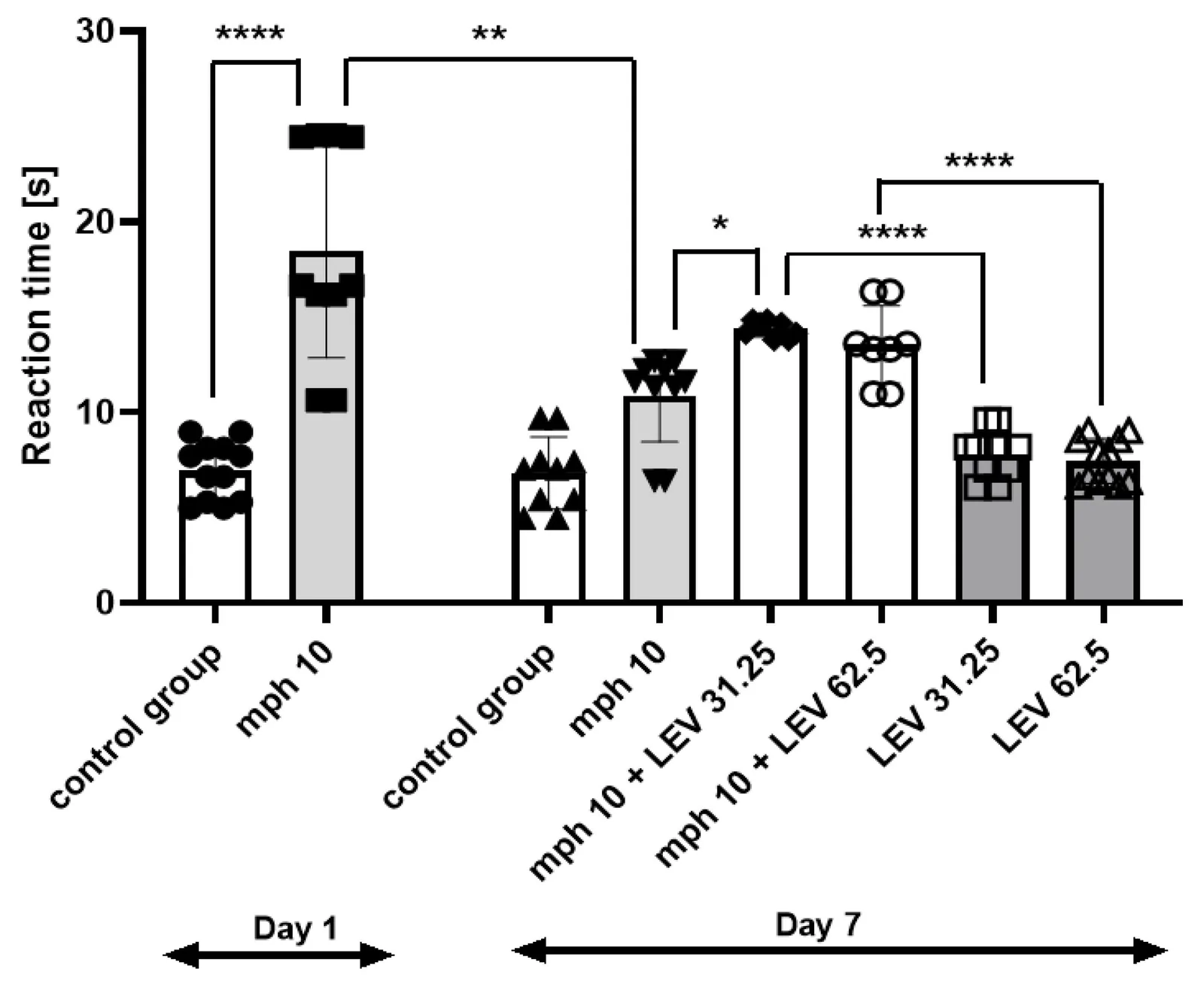
Impact of levetiracetam (LEV) (31.25; 62.5 mg/kg, i.p.) on morphine (MPH) tolerance (10 mg/kg, i.p.) expression in HPT in mice (Swiss). MPH tolerance was obtained by MPH administration for 7 days (twice a day). HPT was carried out on the 1st and 7th days. LEV was administered as a single dose (on day 7), before MPH injection. * *p* < 0.05; ** *p* < 0.01; **** *p* < 0.0001 (Tukey’s test), *N* = 8–10.

In the post hoc test, a statistical prolongation of reaction time was confirmed (*p* < 0.0001) in mice treated with a single MPH dose relative to the control mice. Repeated MPH injections for 7 consecutive days significantly shortened the reaction time of mice (*p* < 0.01) compared with the reaction time recorded after an acute MPH injection. Combination of LEV (31.25 mg/kg) with MPH significantly prolonged the reaction time of animals (*p* < 0.0499) in comparison with animals receiving MPH chronically. However, administration of a higher LEV dose (62.5 mg/kg) did not influence the reaction time. Administration of LEV alone (31.25 and 62.5) did not change the reaction time of mice relative to control animals (Figure 1).

### 3.2. Impact of LEV (31.25; 62.5 mg/kg, i.p.) on MPH (10 mg/kg, i.p.) Tolerance—Acquisition Model

**Figure 2:**
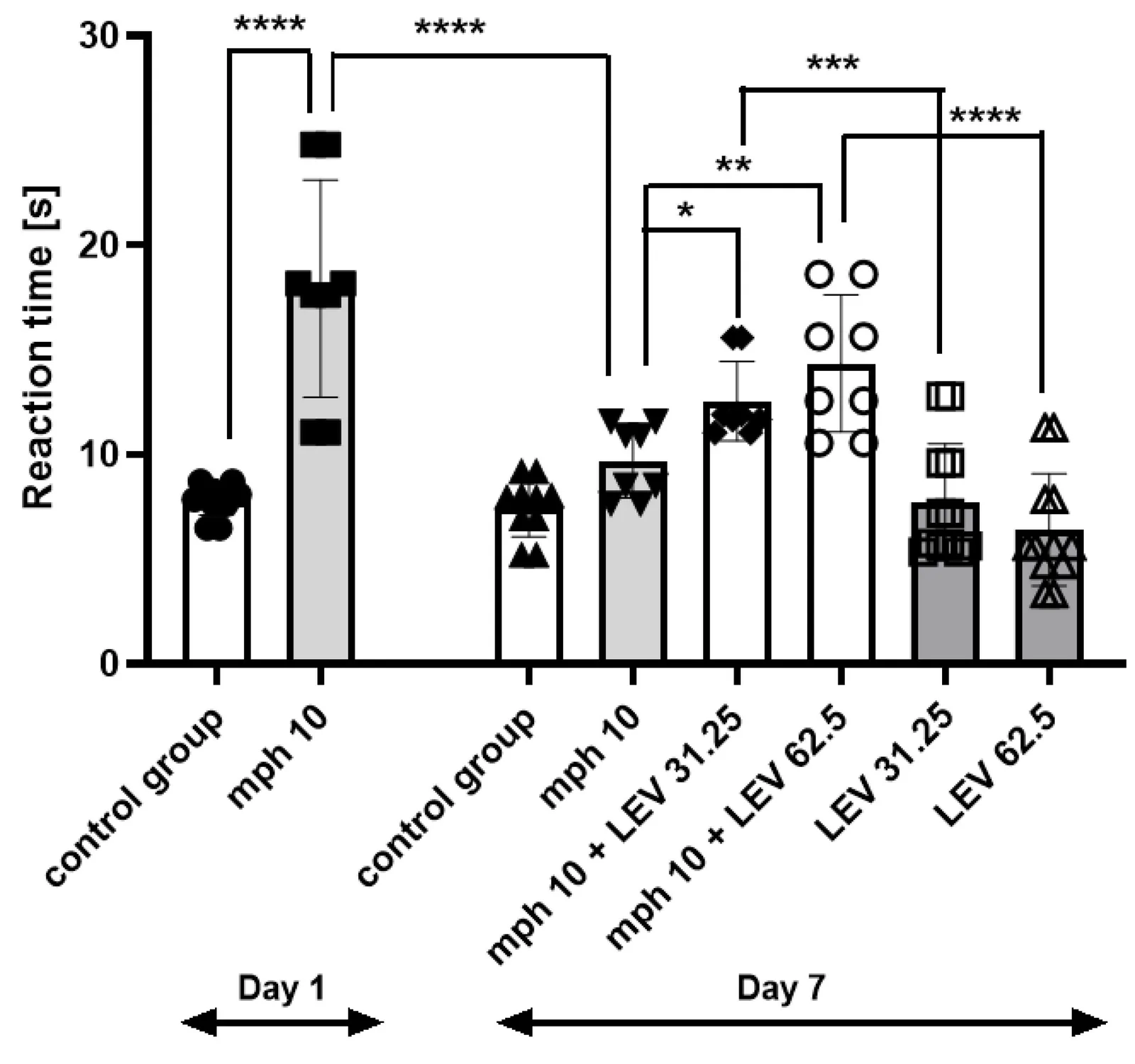
Impact of levetiracetam (LEV) (31.25; 62.5 mg/kg, i.p.) on morphine (MPH) tolerance (10 mg/kg, i.p.) acquisition in HPT in mice (Swiss). HPT was carried out on the 1st and 7th days. LEV was administered from the 1st to the 6th day in the morning. On the last day, only MPH was applied. * *p* < 0.05; ** *p* < 0.01; *** *p* < 0.001; **** *p* < 0.0001 (Tukey’s test), *N* = 8–10.

Two-way ANOVA revealed significant alterations in response latency in animals exposed to both acute and repeated MPH injections, compared with control animals. The following were observed: a day effect (F(1,36) = 17.24; *p* = 0.0002), a drug effect (F(1,36) = 34.97; *p* < 0.0001), and an interaction (F(1,36) = 14.49; *p* = 0.0005). One-way ANOVA confirmed statistical differences in the animals’ response on day 7 (F(5,49) = 15.10; *p* < 0.0001). In the post hoc, acute MPH administration markedly prolonged (*p* < 0.0001) the animals’ response to the stimulus relative to controls. In contrast, chronic MPH application reduced (*p* < 0.0001) the time of animals’ response compared with an acute MPH injection. Chronic treatment with both LEV doses (31.25 and 62.5 mg/kg) significantly prolonged (*p* < 0.019 and *p* < 0.0061, respectively) the time of animals’ response relative to animals receiving chronic MPH. Chronic administration of LEV alone (31.25 mg/kg and 62.5 mg/kg) did not alter the reaction time in mice (Figure 2).

### 3.3. Impact of LEV (31.25; 62.5 mg/kg, i.p.) on NAL-Precipitated (2 mg/kg, i.p.) MPH Withdrawal—Expression Model

**Figure 3:**
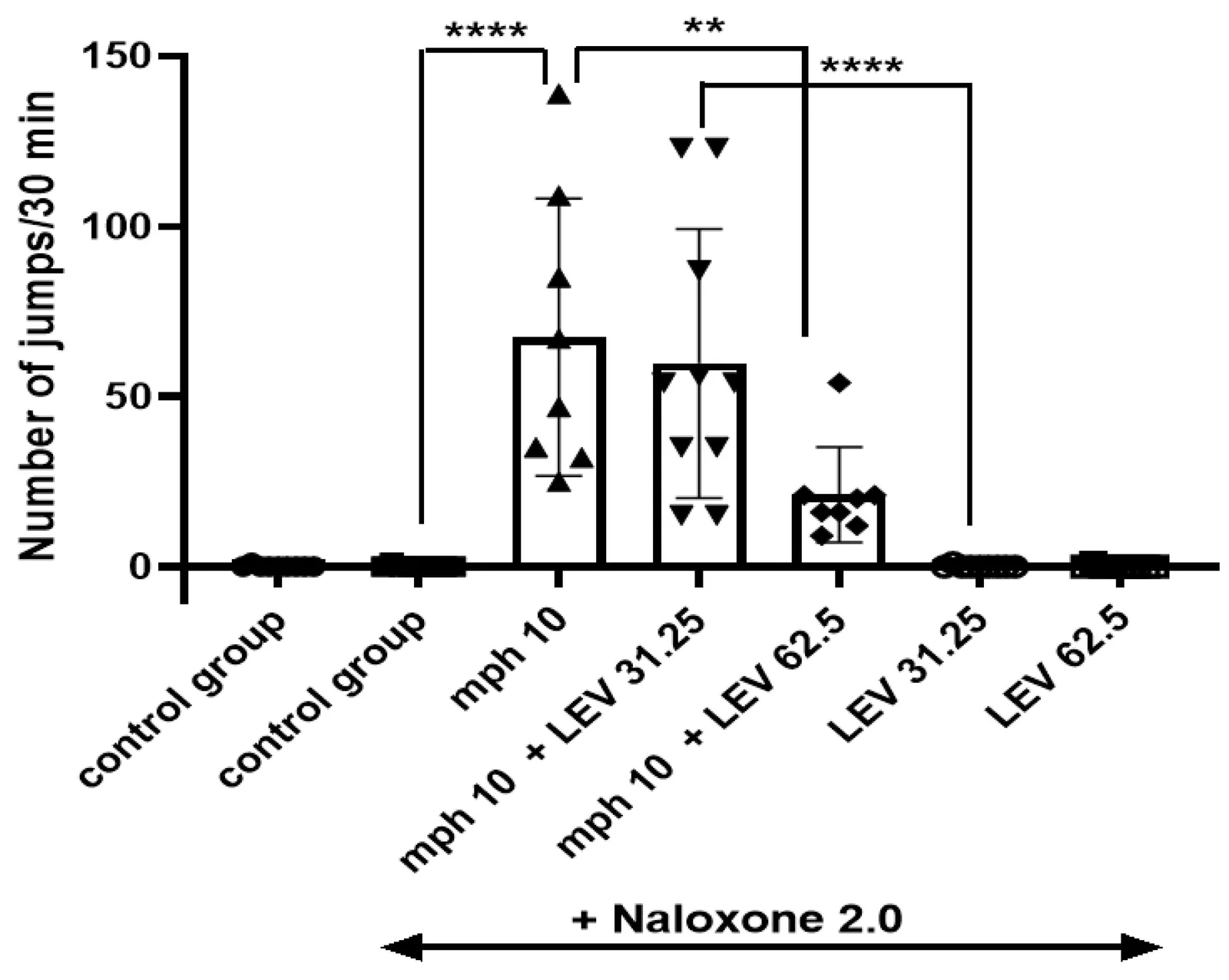
Impact of levetiracetam (LEV) (31.25; 62.5 mg/kg, i.p.) on NAL-precipitated (2 mg/kg, i.p.) morphine (MPH) withdrawal in mice (Swiss)—expression; MPH was administered at increasing doses (from 10 to 50 mg/kg) for 8 days (twice daily). On the 9th day (morning), another MPH dose (50 mg/kg) followed by NAL was given. LEV was administered at a single dose on the last day. ** *p* < 0.01; **** *p* < 0.0001 (Tukey’s test), *N* = 8–10.

One-way ANOVA confirmed statistical differences in MPH-dependent mice that were exposed to a single LEV dose (F(6,55) = 16.2; *p* < 0.0001). Tukey’s test showed that NAL administration in animals repeatedly treated with MPH markedly increased the jumps number (*p* < 0.0001) versus mice not treated with MPH. Administration of a higher LEV dose (62.5 mg/kg) diminished (*p* = 0.002) the number of jumps after NAL exposure. Both doses of LEV alone did not produce an effect on the behavior of animals not exposed to MPH (Figure 3).

### 3.4. Impact of LEV (31.25; 62.5 mg/kg, i.p.) on NAL-Precipitated (2 mg/kg, i.p.) MPH Withdrawal—Acquisition Model

**Figure 4:**
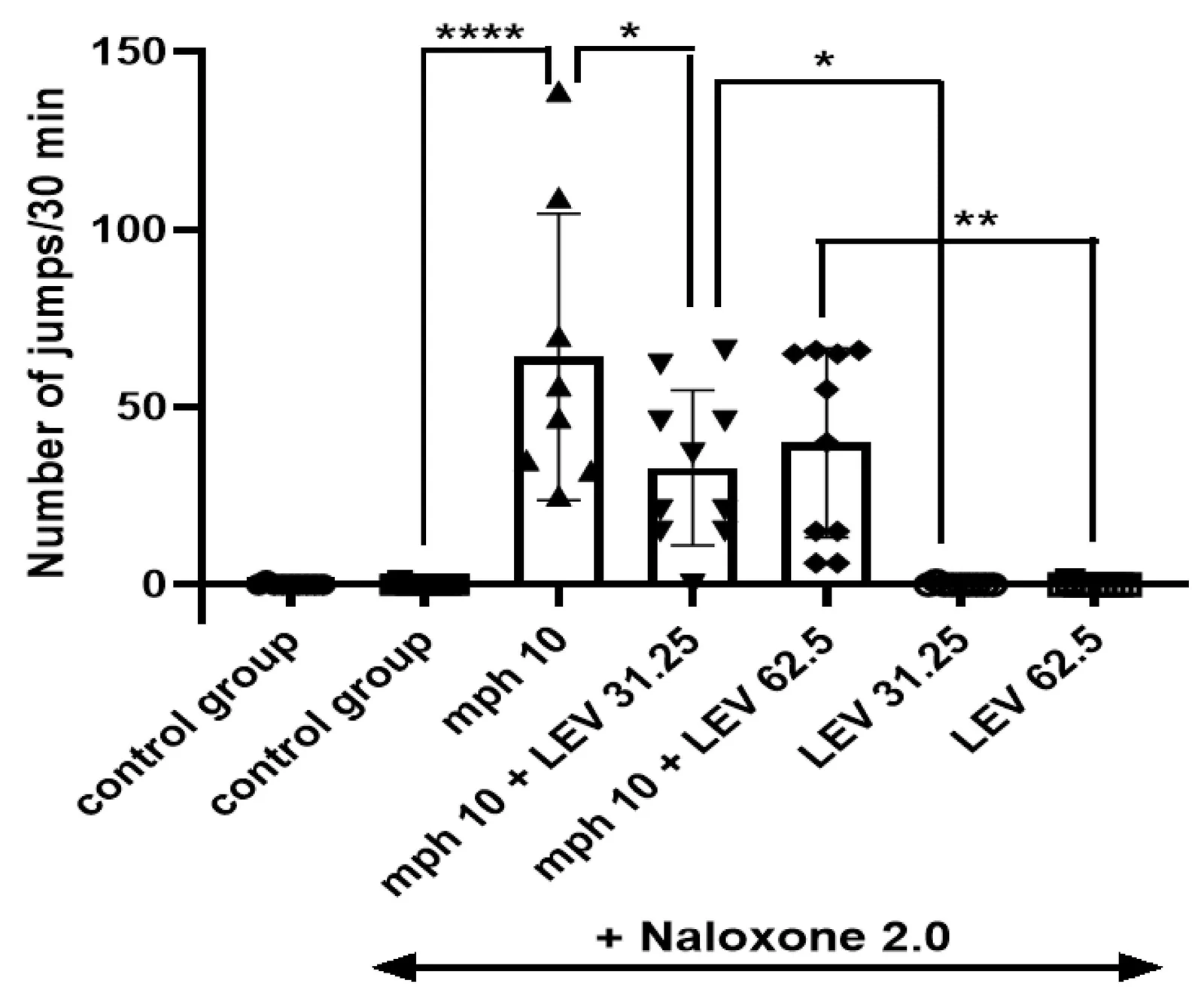
Impact of levetiracetam (LEV) (31.25; 62.5 mg/kg, i.p.) on NAL-precipitated (2 mg/kg, i.p.) morphine (MPH) withdrawal symptoms in mice (Swiss)—acquisition; MPH was administered at increasing doses (from 10 to 50 mg/kg), for 8 days (twice daily). On the 9th day, another dose of MPH (50 mg/kg), followed by NAL, was given. LEV was administered on 1–8 days. * *p* < 0.05; ** *p* < 0.01; **** *p* < 0.0001 (Tukey’s test), *N* = 8–10.

As one-way ANOVA showed, the chronic LEV exposure in MPH-dependent animals affected the severity of jumping behavior (F(6,57) = 15.21; *p* < 0.0001). Tukey’s test revealed that NAL injection in MPH-dependent animals potentiated (*p* < 0.0001) the episodes of jumps in relation to mice not exposed to MPH. The lower dose of LEV (31.25 mg/kg) reduced (*p* = 0.0235) the number of jumps versus MPH-exposed animals. Chronic treatment with LEV at doses of 31.25 and 62.5 mg/kg had no effect on the number of jumps in animals that were not treated with MPH (Figure 4).

### 3.5. Impact of LEV (31.25; 62.5; 125 mg/kg, i.p.) on MPH (10 mg/kg, i.p.) Sensitization—Expression Model

**Figure 5:**
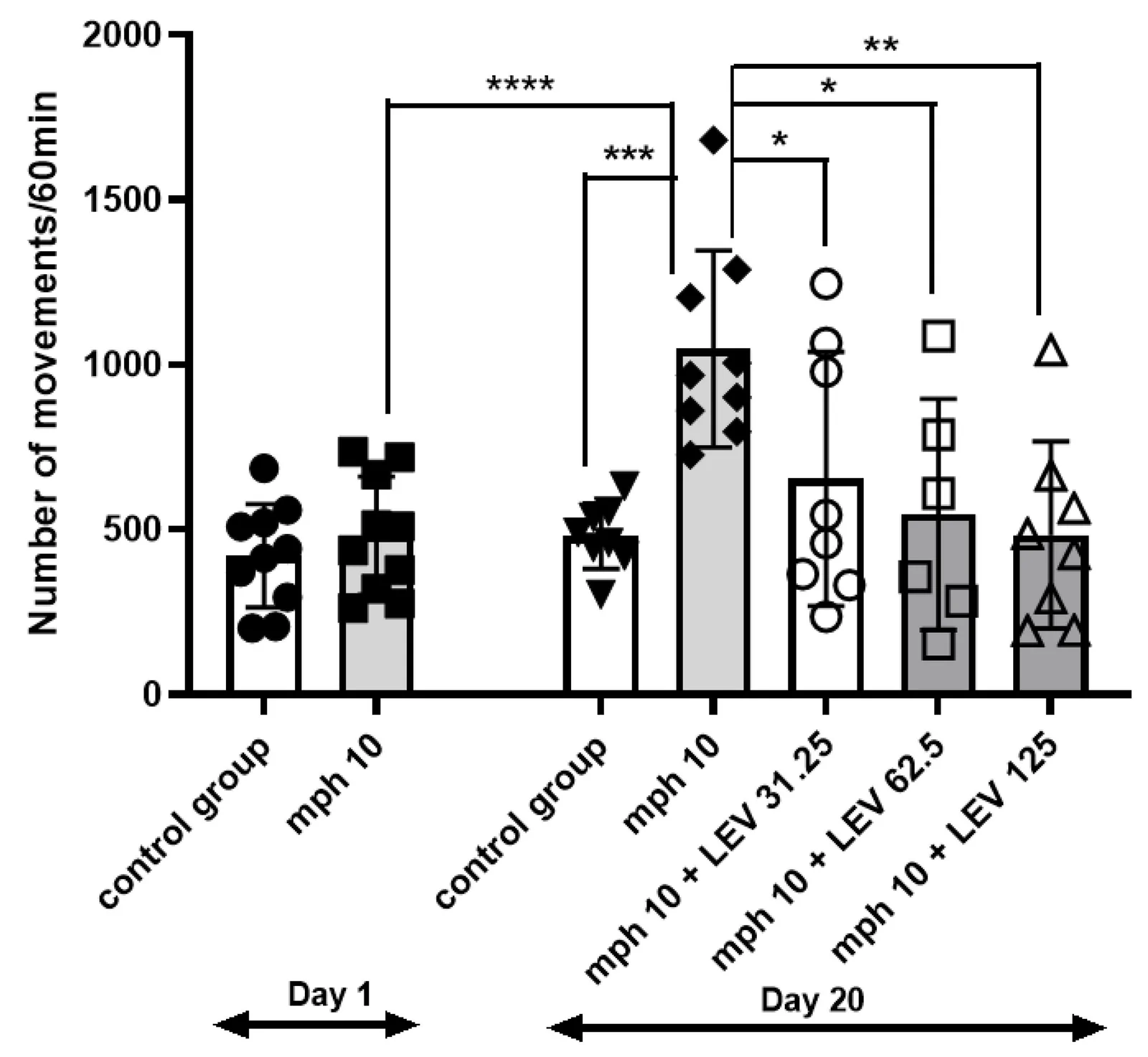
Impact of levetiracetam (LEV) (31.25; 62.5; 125 mg/kg, i.p.) on morphine (MPH) sensitization (10 mg/kg, i.p.) in mice (Swiss)—expression. Behavioral sensitization was achieved by five intermittent MPH injections (every 72 h). After a drug-free period of seven days (on the 20th day), animals received an MPH challenge dose (10 mg/kg). LEV was used at a single dose on the 20th day of the study. * *p* < 0.05; ** *p* < 0.01; *** *p* < 0.001; **** *p* < 0.0001 (Tukey’s test), *N* = 8–10.

One-way ANOVA confirmed alterations in locomotor activity on the 20th day in mice intermittently exposed to MPH and treated with a single LEV dose (F(4,36) = 6.062; *p* < 0.001). On this day, intermittent MPH administration resulted in an increase in locomotor activity of mice versus saline-treated mice (*p* = 0.0018). A single administration of LEV (31.5, 62.5 and 125 mg/kg) during the final testing day in animals receiving intermittent MPH injections significantly reduced locomotor activity (*p* = 0.0183; *p* = 0.0157; *p* = 0.0019, respectively) in relation to MPH-treated mice exposed to the challenge dose (10 mg/kg), Figure 5.

### 3.6. Impact of LEV (31.25; 62.5; 125 mg/kg, i.p.) on MPH (10 mg/kg, i.p.) Sensitization—Acquisition Model

Based on analysis of variance, it was shown on the last day that an MPH challenge dose evoked alterations in the locomotor activity of mice receiving intermittent injections of MPH and LEV on days 1, 4, 7, 10, and 13 (F(4,37) = 10.02; *p* < 0.0001).

**Figure 6:**
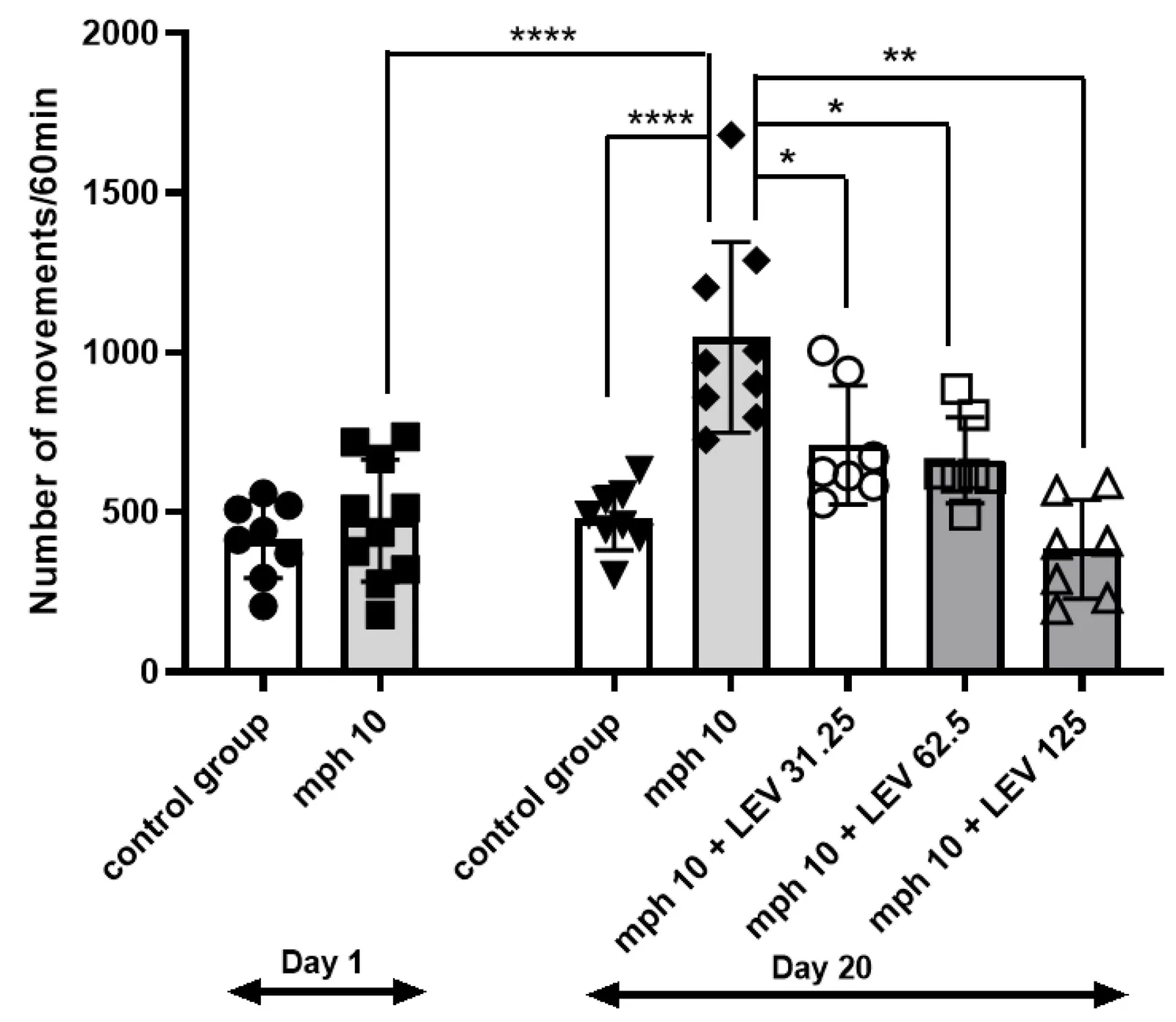
Impact of levetiracetam (LEV) (31.25; 62.5; 125 mg/kg, i.p.) on morphine (MPH) sensitization (10 mg/kg, i.p.) in mice (Swiss)—acquisition. Behavioral sensitization was achieved by five intermittent MPH injections (every 72 h). After a drug-free period of seven days (on the 20th day), animals received an MPH challenge dose (10 mg/kg). Five injections of LEV were administered on days 1, 4, 7, 10, and 13. * *p* < 0.05; ** *p* < 0.01; **** *p* < 0.0001 (Tukey’s test), *N* = 8–10.

*Post hoc* comparison demonstrated a potentiation of locomotor activity in animals challenged with an MPH dose versus control mice (on day 20) (*p* = 0.0001). Repeated LEV administration (31.25, 62.5, and 125 mg/kg) together with MPH on days 1, 4, 7, 10, and 13 significantly reduced the animals’ locomotor activity on the final testing day (day 20) (*p* = 0.0302, *p* < 0.0244, and *p* < 0.01, respectively) compared with the locomotor activity observed in mice receiving MPH alone, Figure 6.

## 4. Discussion

The major result presented in this paper is that LEV, a second-generation anticonvulsant, has an influence on MPH dependence-related behaviors in mice. It was shown that LEV was able to inhibit MPH tolerance to the antinociceptive effect. Moreover, acute and repeated LEV exposure effectively diminished the intensity of MPH withdrawal and attenuated MPH-induced sensitization. LEV was used at doses of 31.25, 62.5, and 125 mg/kg. The dose selection was based on the available literature data [27,28] and on preliminary experiments presented in Supplementary File S1. Previous studies in mice have used LEV within a comparable dose range, including doses close to 30–60 mg/kg [27,28]. In our preliminary experiments, LEV at the selected doses did not influence spontaneous locomotor activity, motor coordination assessed in the rotarod test, or myorelaxant-like effects and muscle tone evaluated in the chimney test. Therefore, these doses were considered appropriate for subsequent behavioral experiments, as they were not associated with nonspecific motor impairment, sedation, or myorelaxant-like effects that could interfere with the interpretation of the results.

Another important issue in the study is a risk of interactions between LEV and MPH. Although the mechanisms of both compounds are completely different, pharmacodynamic interactions between them cannot be ruled out because both drugs act on the central nervous system. Thus, their administration at higher doses may cause dizziness or sedation. In our preliminary study, no significant effect on locomotor activity in mice was observed when a combination of the two drugs was administered (data were not published). Moreover, LEV does not affect cytochrome P450 enzymes, which are often involved in drug–drug interactions. MPH is predominantly metabolized by hepatic glucuronidation to MPH-3-glucuronide and MPH-6-glucuronide, whereas CYP-mediated metabolism is not the main metabolic pathway for MPH. LEV is not a P-glycoprotein inducer, which may also reduce the risk of interaction during absorption [38]. Thus, the lack of pharmacokinetically significant interactions may make LEV a useful option in polytherapy.

MPH dependence and addiction-related behaviors may be assessed using several experimental paradigms. We focused on three well-established models: MPH tolerance to MPH-induced antinociception, NAL-precipitated MPH withdrawal, and MPH-induced sensitization. The antinociceptive tolerance model, assessed using the HPT, was chosen as one of the most clinically relevant and extensively characterized consequences of repeated opioid exposure. Jumping behavior was selected as the main withdrawal endpoint because it represents a robust, reproducible, and easily quantifiable sign of MPH withdrawal in rodents and is commonly used as an index of the intensity of physical dependence on morphine. Other somatic or autonomic withdrawal signs, like wet-dog shakes, teeth chattering, or body weight loss, were not assessed in this study. Locomotor sensitization was selected as a widely used, well-described model reflecting drug-induced behavioral neuroadaptations after repeated MPH administration. Although other models of tolerance, withdrawal, and sensitization are available, these paradigms were chosen because they are widely used in behavioral pharmacology, suitable for pharmacological screening, and allow reliable and standardized assessment under our experimental conditions.

In the first part of this study, the LEV activity on MPH tolerance in the HPT was assessed in two schedules: expression and acquisition. For this purpose, a typical experimental model was used [39,40]. MPH tolerance was obtained by repeated administration for 7 consecutive days at a dose of 10 mg/kg. The HPT was carried out on days 1 and 7. As observed on the final day, a reduction in the animals’ response to the thermal stimulus was noticed, which confirmed MPH tolerance. Both acute and chronic doses of LEV attenuated MPH tolerance in mice. After acute LEV exposure in the expression model, MPH tolerance was inhibited after application of the lower dose. Meanwhile, repeated exposure to both LEV doses, in the acquisition model, reduced MPH tolerance in two cases. It should be emphasized that our results correspond with earlier observations [41] in which significantly higher LEV doses (60, 300, or 900 mg/kg, i.p.) were used, and MPH was administered at a high dose of 50 mg/kg for 3 days (once a day). They observed tolerance in another test, the tail flick test. These authors documented that LEV at doses of 300 and 900 mg/kg effectively inhibited the development of MPH tolerance, while the expression of MPH tolerance was reduced when the highest LEV dose was injected in mice. Thus, these results show that LEV is able to attenuate MPH tolerance in animal models.

There is also a study showing that rat exposure to a higher LEV dose—564 mg/kg/day—was effective in reducing seizures, but this activity was short-term despite maintained LEV levels in serum and brain [42]. This suggests that chronic exposure to LEV alone may lead to the development of pharmacodynamic tolerance. This effect was not observed in our study because there were no alterations in the animals’ reaction after repeated exposure to LEV alone (Figure 2).

Currently, it is believed that desensitization of opioid µ receptors is a crucial mechanism underlying MPH tolerance. This process is related to higher adenylate cyclase activity and an increase in cAMP levels in various brain areas, including the periaqueductal gray (PAG) [43]. Increased cAMP levels in the PAG, a structure belonging to descending pain pathways, increase presynaptic GABA release and the expression of GABAA receptors [44]. Another mechanism associated with the desensitization process is increased activation of NMDA receptors in postsynaptic membranes [45], which co-occur with µ receptors in nociceptive pathways, such as the dorsal horns, PAG and other structures. It is believed that signals resulting from µ receptor activation stimulate NMDA receptors, leading to increased nitric oxide release and attenuation of µ-opioid receptor activity [46,47,48]. Moreover, AMPA receptors in descending pain pathways may also participate in desensitization [49]. Glutamatergic neurons located in the PAG send projections to the RVM and act on AMPA receptors that coexist with GABAergic terminals. As a result, transmission in the dorsal horns of the spinal cord is modified [49]. Other intracellular mechanisms may also be responsible for MPH tolerance, such as endocytosis of opioid receptors [50], deregulation of β-arrestin levels inside the cells [51,52,53], or alterations in the activity of MAP kinases, e.g., ERK1/2 [54]. Considering the complex LEV-induced mechanisms and its interaction with SV2A protein, which is also present on glutamatergic terminals, it may be hypothesized that synaptic modulation could be related to the attenuation of MPH tolerance observed in our study. However, this explanation remains speculative, as no molecular or neurochemical analyses were performed.

In the second group of experiments, the influence of acute and chronic LEV administration on MPH withdrawal was assessed. A standard model of MPH withdrawal was applied, in which mice received MPH at doses ranging from 10 to 50 mg/kg for 9 consecutive days, and NAL (2 mg/kg) was used to induce withdrawal symptoms. In contrast to several previously described protocols employing more intensive MPH exposure, typically based on escalating MPH doses administered over 2–4 days and higher NAL doses of 4–6 mg/kg to precipitate withdrawal [33,55,56], the model used in the present study involved a relatively prolonged MPH administration schedule with gradually increasing doses and a lower NAL dose of 2 mg/kg. Importantly, this procedure induced a clear withdrawal response while keeping mortality during MPH exposure at a very low level. It was shown that both single and repeated exposure to LEV reduced the MPH withdrawal signs manifested as jumps. To date, research on LEV in MPH dependence is extremely limited.

The MPH withdrawal symptoms develop as a consequence of reduced dopamine levels in the nucleus accumbens [57]. Other neurotransmitters in the brain are also involved in this phenomenon, such as increased glutamate release [58], increased noradrenaline release [59], and decreased GABA secretion [60]. In addition, changes in intracellular signaling have been observed, including an increase in cAMP levels [61] and modification of MAP kinases (for example, ERK ½) [62,63].

Finally, the influence of LEV on MPH sensitization was studied. The sensitization developed as a consequence of intermittent administration of a subeffective MPH dose—10 mg/kg. After a drug-free period (7 days), an MPH challenge dose (10 mg/kg) was injected [34,35,64]. It was shown that the MPH challenge dose significantly intensified the locomotor activity in mice, confirming the appearance of behavioral sensitization in the studied animals. Moreover, both single and chronic exposure to LEV reduced this locomotor activity. LEV-induced attenuation of MPH sensitization might be associated, at least in part, with modulation of GABAergic transmission [65]; however, this hypothesis requires further experimental confirmation. MPH-induced behavioral sensitization in rodents is considered a preclinical model of drug-induced neuroadaptations that may be relevant to enhanced drug “wanting” and relapse vulnerability; however, it should not be interpreted as a direct measure of craving in humans [21,66,67]. Therefore, the observed effects of LEV should be interpreted as evidence of LEV’s ability to modulate MPH-induced behavioral sensitization, rather than as direct evidence of anti-craving activity. Further studies are required to confirm its pharmacological relevance, underlying mechanisms, and potential therapeutic applicability. To the best of our knowledge, these results are novel, as there are currently no reports showing an influence of LEV on MPH-induced behavioral sensitization.

Behavioral sensitization is predominantly related to increased dopamine secretion in the structures of the mesolimbic system [21,66,67], which is manifested, among other effects, by increased locomotor activity in animals. Moreover, adaptive changes develop in dopaminergic and glutamatergic structures of the mesolimbic system [68,69,70,71], including changes in both NMDA glutamate receptors [72] and AMPA receptors [73]. Additionally, increased GABA release in the hippocampus has also been observed during behavioral sensitization [74], while stimulation of GABAergic neurons reduced sensitization in rats [75]. It may be hypothesized that the inhibitory effect of LEV on behavioral sensitization may be associated with its indirect effect on GABAergic transmission. However, this hypothesis requires further studies for confirmation.

The activity of LEV observed in our study was not consistently dose-dependent across all behavioral paradigms. This was particularly evident in the expression of tolerance and in the acquisition of withdrawal signs. Such findings may reflect a non-linear or endpoint-specific pharmacological response, which is frequently observed in behavioral studies involving complex neurobiological processes. Moreover, the lack of a fully consistent dose-dependent pattern may be explained by the complexity of the experimental protocol used in our study. Lower doses of LEV may be effective under some experimental conditions, whereas higher doses may be more effective under others. Differences between the acquisition and expression phases of MPH-induced adaptations may also be important, as these phases may involve partially distinct neurobiological mechanisms and may therefore differ in their sensitivity to LEV. In addition, ceiling effects or behavioral variability cannot be excluded, particularly in these complex models. However, since LEV at the selected doses did not produce nonspecific motor impairment, impaired coordination, or myorelaxant-like effects in preliminary tests, the observed effects are unlikely to result solely from general behavioral suppression. Therefore, the present outcomes suggest that LEV modifies selected MPH-induced behavioral responses rather than demonstrating a classical dose–response relationship.

The present study has some weaknesses, including a methodological approach assessing behavioral effects of LEV without identifying any precise molecular mechanisms underlying these effects. LEV selectively interacts with SV2A protein and may theoretically influence synaptic transmission; however, these pathways were not experimentally explored in this study. Similarly, glutamatergic, GABAergic, and dopaminergic transmission, NMDA receptor function, and ERK signaling were not directly evaluated. Therefore, the proposed mechanisms should be regarded as hypothetical. Future studies are necessary to identify molecular and neurochemical mechanisms underlying the effects of LEV in MPH dependence.

A second limitation of our study is the narrow scope of observed MPH withdrawal signs. Only jumps were assessed as the main quantitative endpoint. Other withdrawal signs were also observed in these mice; however, they were not recorded. Therefore, future studies should include a broader panel of somatic and autonomic withdrawal signs to provide a more comprehensive evaluation of withdrawal severity.

Another limitation is that we did not perform an analogous study in female mice. Some sex-related differences in the efficacy of LEV have been observed in humans [76]. Females generally show decreased sensitivity to opioids compared with males, due to variations in opioid receptor density and function influenced by sex hormones like estrogen and testosterone. Thus, the present findings apply only to male mice and should not be generalized to females. For better recognition of this issue, future studies should include female animals to determine whether LEV effects on MPH tolerance, MPH withdrawal, and MPH-induced behavioral sensitization are sex-dependent.

Furthermore, our study did not include positive pharmacological control groups for each behavioral paradigm, such as clonidine in the NAL-precipitated withdrawal model or memantine in the MPH tolerance model. Their inclusion would allow a more precise assessment of the relative magnitude of LEV’s effects. Therefore, future studies should include appropriate comparator drugs to better define the efficacy of LEV in MPH dependence models.

Taken together, additional studies are required to determine the therapeutic and mechanistic relevance of LEV in MPH dependence.

## 5. Conclusions

In conclusion, this study demonstrates that LEV attenuates selected behavioral manifestations associated with repeated MPH exposure in mice, including the development of tolerance to MPH-induced antinociception, NAL-precipitated withdrawal signs, and MPH-induced behavioral sensitization. Particular attention should be paid to the beneficial effect of LEV on MPH sensitization, as this finding provides, to the best of our knowledge, one of the first preclinical indications that LEV may modulate sensitization-related behavioral adaptations after repeated MPH administration. These results suggest that LEV may influence selected behavioral adaptations related to MPH dependence in preclinical experimental models.

However, the present data should be interpreted as evidence of a preclinical behavioral effect and do not provide direct evidence of therapeutic efficacy or clinical applicability in humans. Moreover, this study does not include mechanistic evidence for the participation of SV2A protein or other molecular pathways. Therefore, further studies are necessary to verify the potential involvement of SV2A-dependent mechanisms, glutamatergic/GABAergic transmission, and dopaminergic signaling in the effects of LEV on MPH dependence-related behaviors.

## Data Availability

The datasets supporting the results presented in this study may be obtained from the authors upon reasonable request.

## Supplementary Materials

The following supporting information can be downloaded at: https://www.mdpi.com/article/10.3390/life16081274/s1, Supplementary File S1: Preliminary behavioral assessment of levetiracetam; Supplementary File S2: Summary of experimental design and group allocation.
