# Supplementary material for: The Impact of Levetiracetam on Various Symptoms of Morphine Dependence in Mice
Source: Life (Basel). 2026 Jul 31;16(8):1274. doi: 10.3390/life16081274 (PMC13514814; doi:10.3390/life16081274)
Supplement: Supplementary file 1 [file life-16-01274-s001.zip › Supplementory File S1.pdf]

The effects of acute exposure to levetiracetam (LEV: 31,5; 62,5; 125; 250 mg/kg, ip) on locomotor activity, motor coordination and miorelaxation in mice

a) The locomotor activity test

Locomotor activity was measured using actimeters (Multiserv, Lublin, Poland). The apparatus is consisted of circular metal cages (32 cm in diameter, equipped with photoresistor cells. Each interruption of a light beam by a moving animal was automatically recorded by a counter as one locomotor movement. The number of beam interruptions was used as an index of spontaneous locomotor activity. Levetiracetam or vehicle in control group was administered 30 min before the test. Each mouse was placed individually in the apparatus, and locomotor activity was recorded for 60 min.

b) The rotarod test

Motor coordination was assessed using the rotarod test. The ability of mice to maintain balance and coordinated movement on a rotating rod was studied. before the experiment, the animals were trained for three consecutive days for up to 3 min, with an unlimited number of trials allowed for each animal. The rotation speed was kept constant at 16 rpm. On the test day, the latency to fall from the rotating rod was recorded, with a maximum cut-off time of 60 s. When an animal fell from the rod, the measurement was automatically stopped and the latency to fall was recorded by the apparatus. Levetiracetam or vehicle in control group was administered 60 min before the test.

c) The chimney test

Muscle relaxation was assessed using the chimney test. The apparatus consisted of a vertically oriented tube, referred to as chimney, with a rough internal surface and constant dimensions: 25 cm in length and 3 cm in external diameter. At the beginning of the test, each mouse was introduced into the horizontally positioned chimney. When the animal reached the opposite end, the chimney was turned to a vertically position, allowing the mouse to escape by climbing backwards. On the day of testing, the time required for each mouse to leave the chimney was measured, with a maximum cut-off time of 60 s. Levetiracetam or vehicle was administered 60 min before the muscle relaxation test.

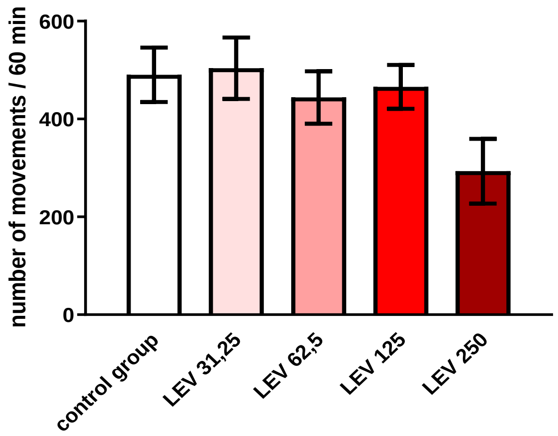

Figure 1. Effect of levetiracetam (LEV) (31.5; 62.5; 125; 250 mg/kg, ip) on the mobility of mice assessed in the locomotor activity test; N=8-10.

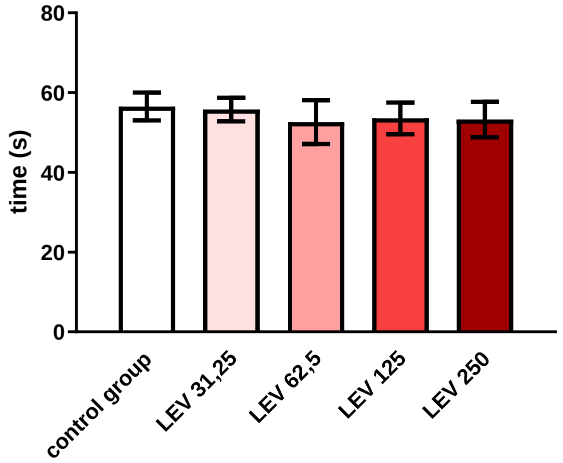

Figure 2. Effect of levetiracetam (LEV) (31.25; 62.5; 125; 250 mg/kg, ip) on motor coordination in mice assessed by the rota-rod test; N=8-10.

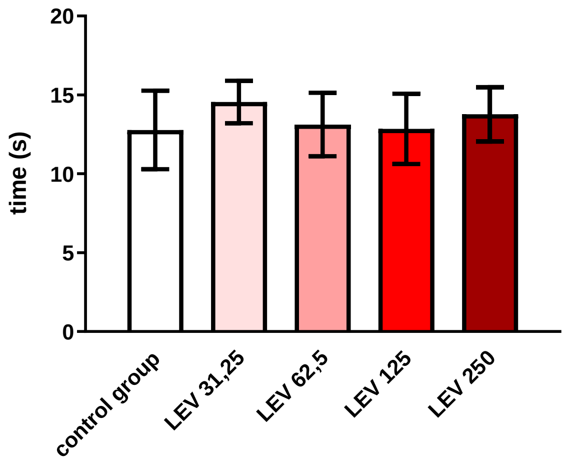

Figure 3. Effect of levetiracetam (LEV) (31.25; 62.5; 125; 250 mg/kg, ip) on myorelaxation in mice assessed by the chimney test; N=8-10.

One-way ANOVA revealed that acute administration of levetiracetam at doses of 31.25, 62.5, 125, and 250 mg/kg did not produce statistically significant differences in spontaneous locomotor activity [$F(4,41) = 2.027$; $p = 0.1085$] (Figure 1), motor coordination assessed in the rotarod test [$F(4,41) = 0.1644$; $p = 0.9552$] (Figure 2), or muscle relaxation evaluated in the chimney test [$F(4,39) = 0.1286$; $p = 0.9711$] (Figure 3). These findings suggest that the tested doses of levetiracetam were not associated with nonspecific motor impairment, impaired coordination, or myorelaxant activity.
