# Supplementary material for: The Impact of Levetiracetam on Various Symptoms of Morphine Dependence in Mice
Source: Life (Basel). 2026 Jul 31;16(8):1274. doi: 10.3390/life16081274 (PMC13514814; doi:10.3390/life16081274)
Supplement: Supplementary file 1 [file life-16-01274-s001.zip › Supplementory File S2.pdf]

Table 1. Summary of experimental design and treatment schedule.

**Morphine antinociceptive tolerance**					
**GROUP**	**N**		**ADMINISTERED DRUGS AND DOSES [mg/kg]**	**Testing day**	**Analyzed endpoint**
	**expression**	**acquisition**			
Control	10	10	0.5% methylcellulose solution	1	hot-plate latency
Morphine	10	8	morphine hydrochloride 10		
Control	9	10	0.5% methylcellulose solution	1 and 7	
Morphine	10	8	morphine hydrochloride 10		
Morphine + LEV 31.25	10	9	morphine hydrochloride 10<br>levetiracetam 31.25		
Morphine + LEV 62.5	10	8	morphine hydrochloride 10<br>levetiracetam 62.5		
LEV 31.25	9	10	levetiracetam 31.25		
LEV 62.5	10	10	levetiracetam 62.5		
**Naloxone-precipitated withdrawal**					
**GROUP**	**N**		**ADMINISTERED DRUGS AND DOSES [mg/kg]**	**Testing day**	**Analyzed endpoint**
	**expression**	**acquisition**			
Control	9	9	0.5% methylcellulose solution	9	Number of jumps / 30 min
Control	9	9	0.5% methylcellulose solution<br>naloxone hydrochloride 2		
Morphine	8	8	morphine hydrochloride 10-50		
Morphine + LEV 31.25	10	10	morphine hydrochloride 10-50<br>naloxone hydrochloride 2<br>levetiracetam 31.25		
Morphine + LEV 62.5	8	10	morphine hydrochloride 10-50<br>naloxone hydrochloride 2<br>levetiracetam 62.5		
LEV 31.25	9	9	levetiracetam 31.25		
LEV 62.5	9	9	levetiracetam 62.5		
**Morphine-induced locomotor sensitization**					
	**N**		**ADMINISTERED DRUGS AND DOSES [mg/kg]**	**Testing day**	**Analyzed endpoint**
	**expression**	**acquisition**			
Control	10	8	0.5% methylcellulose solution	1	Locomotor activity / 60 min
Morphine	10	10	morphine hydrochloride 10		
Control	8	8	0.5% methylcellulose solution	20	
Morphine	9	9	morphine hydrochloride 10		
Morphine + LEV 31.25	8	8	morphine hydrochloride 10<br>levetiracetam 31.25		
Morphine + LEV 62.5	8	8	morphine hydrochloride 10<br>levetiracetam 62.5		
Morphine + LEV 125	8	9	morphine hydrochloride 10<br>levetiracetam 125		
